# Good subjective outcome and low risk of revision surgery with a novel customized metal implant for focal femoral chondral lesions at a follow-up after a minimum of 5 years

**DOI:** 10.1007/s00402-021-04160-z

**Published:** 2021-09-14

**Authors:** Mohanad Al-Bayati, Nicolas Martinez-Carranza, David Roberts, Magnus Högström, Anders Stålman

**Affiliations:** 1 Orthopedic Department, Skövde Hospital, Skövde, Sweden; 2 Department of Orthopedics, Institution of Clinical Sciences, Intervention and Technology (CLINTEC), Karolinska Institutet, Stockholm, Sweden; 3 Division of Orthopedics, Karolinska University Hospital, Stockholm, Sweden; 4 Ortho Center Skåne and Department of Orthopedics, Skåne University Hospital, Malmö, Sweden; 5 Department of Surgical and Perioperative Sciences, Sports Medicine Umeå and Orthopedics, Umeå University, Umeå, Sweden; 6 MMK, Stockholm Sports Trauma Research Center, Karolinska Institutet, Solna, Sweden; 7 Capio Artro Clinic, Sophiahemmet, Stockholm, Sweden

**Keywords:** Focal cartilage injuries, Prosthetic inlay resurfacing, Osteochondral injury, Knee

## Abstract

**Background and purpose:**

Patients with focal cartilage lesions experience functional impairment. Results for biological treatments in the middle-aged patient is poor. Previous studies with focal prosthetic inlay resurfacing have shown a higher risk of conversion to total knee replacement at mid-term follow-up. A novel customized implant (Episealer, Episurf, Stockholm, Sweden) has been proposed to improve implant positioning and survival. The primary objective was to assess subjective-, objective function and implant survival at a minimum of five years after surgery.

**Materials and methods:**

The inclusion criteria were patients aged 30–65 years with symptomatic focal chondral defects in the medial femoral condyle, International Cartilage Research Society grade 3 or 4 and failed conservative or surgical treatment. Minimum follow-up of 5 years. Clinical and radiologic assessments were made. Patient-reported outcome measurements at the latest follow-up were compared with the baseline data for the Knee injury and Osteoarthritis Outcome Score (KOOS), the EuroQoL (EQ-5D), the Tegner Activity Scale and a Visual Analog Scale of pain (VAS 0–10).

**Results:**

Ten patients with the mean follow-up period of 75 months (60–86 months, SD 10) were included. Signs of osteoarthritis were seen in one patient (Ahlbäck 1). No cases with revision to knee replacement. VAS for pain and KOOS showed improvements that reached significance for VAS (*p* ≤ 0.001) and the KOOS subscores Pain (*p* = 0.01), ADL (*p* = 0.003), Sport and Recreation (*p* = 0.024) and Quality of Life (*p* = 0.003).

**Conclusion:**

A good subjective outcome, a low risk of progression to degenerative changes and the need for subsequent surgery were seen at the mid-term follow-up with this customized focal knee-resurfacing implant.

**Level of evidence:**

Prospective case series, level 4.

## Introduction

Participation in sport as a competitive or recreational athlete is associated with a high incidence of cartilage injuries due to traumatic or chronic repetitive damage [1, 2]. Patients with focal cartilage lesions often experience significant functional impairment, impaired quality of life and pain to the same extent as patients with severe osteoarthritis [3]. The cartilage lacks innate abilities to mount a regenerative response to injury and even a small chondral lesion could induce unicompartmental osteoarthritis [4]. The natural history of the isolated chondral defect is not completely understood, but it is clear that patients with untreated focal cartilage injuries are more prone to experience a progression of cartilage damage, eventually leading to osteoarthritis [5]. Biological treatments, such as microfracture and autologous chondrocyte implantation, have the potential for cartilage healing and pain relief in the young patient. However, the treated defects might degenerate with time and results for middle-aged and older patients are poor [6, 7]. For the active middle-aged patient with a symptomatic cartilage defect, the treatment options are few. Knee arthroplasties in this patient category run a high risk of failure [8].

Focal prosthetic inlay resurfacing has been proposed as a bridge between biological treatment and conventional joint arthroplasty [9]. A promising short-term outcome is described, but a high rate of revision to knee arthroplasty has been reported at mid-term follow-ups [10]. It has been suggested that more accurate implant positioning would enhance implant survival [11]. The development of a customized prosthesis and guide system designed precisely to fit the cartilage defect in location and size has the potential to improve implant positioning and thereby avoid damage to the opposing cartilage [12]. Two-year clinical results with this implant (Episealer, Episurf AB, Stockholm, Sweden) and follow-up with Radio Stereometric Analysis (RSA) showed no implant migration and a good subjective outcome [13]. A multicenter study with 75 patients operated on with the implant that were evaluated at a minimum of 24 months after surgery demonstrated a low failure rate of 2.5% and clinically significant improvements in pain and function scores [14]. Mid-term follow-up of 30 patients showed good implant safety and lower risk of revision surgery [15]. There is no long-term follow-up presented for this implant with full clinical and radiological assessment.

The primary objective in this study was to assess the subjective and objective outcome at a minimum of 5 years with this customized focal knee-resurfacing implant. We hypothesize that a good subjective outcome is preserved and that the risk of osteoarthritis development and the need for revision to knee arthroplasty is low.

## Materials and methods

The inclusion criteria were patients aged 30–65 years with symptomatic focal chondral defects of the central medial femoral condyle, International Cartilage Research Society (ICRS) grade 3 or 4 diagnosed with MRI and verified with arthroscopy. All the patients had previously experienced the failure of conservative or surgical treatment with persistent pain and disability, such as drilling or microfracture. Patients with a BMI of > 35 kg/m^2^, unaddressed instability or concomitant injuries, such as meniscus injuries, apart from minor flap tears with an intact rim, signs of more general cartilage degeneration or established osteoarthritis, were excluded.

A double-coated Ti–HA monobloc Cr–Co femoral condyle implant together with specific guide instruments, manufactured from MRI data using the computer-aided design/computer-aided manufacturing (CAD/CAM) technique, was implanted (Episealer, Episurf AB, Stockholm, Sweden). The implant, surgical technique and post-op protocol have previously been described [13].

At a follow-up after a minimum of 5 years, clinical and radiologic assessments were made. Signs of effusion and reduced range of motion compared with the contralateral knee were evaluated. Standing weight-bearing radiographs were taken in the antero-posterior and lateral views and evaluated according to Ahlbäck’s classification of osteoarthritis [16]. Patient-reported outcome measurements (PROMS) at the latest follow-up were compared with the baseline and 2 year post-op data for the Knee injury and Osteoarthritis Outcome Score (KOOS), the EuroQoL (EQ-5D), the Tegner Activity Scale and a Visual Analog Scale of pain (VAS 0–10).

All the data were analyzed using IBM SPSS Statistics for Mac, version 25 (IBM Corp, Armonk, NY, USA). Data for patient demographics and patient-related outcome measurements are expressed as the mean and standard deviation or median and range depending on the category of data. Group differences were analyzed with the Mann–Whitney rank sum test, two-tailed. A *p* value of less than 0.05 was considered statistically significant.

The study was approved by the Ethics Committee 2012/109-3171 and 2019-03204.

## Results

Ten patients, three females and seven males, fulfilled the inclusion criteria, were included after informed consent, and were all available at follow-up. The mean follow-up period was 6 years and 3 months (75 months, range 60–86 months, SD 10). All the procedures were performed on the medial femoral condyle (Table 1).

**Table 1:** Demographics

Patients	Age (years)	Gender	ICRS (1–4)	Involved knee	Implant size (mm)	Localization	Previous surgery	ROM healthy knee (extension-flexion)	ROM operated knee
1	64	Male	4	Left	20	Medial condyle	Microfracture	0 to 135	0 to 135
2	50	Female	4	Right	17	Medial condyle	ACL rec. minor medial meniscus resection	0 to 140	0 to 140
3	44	Female	4	Left	20	Medial condyle	ACL rec. minor medial meniscus resection Microfracture	− 5 to 135	0 to 150
4	57	Male	4	Right	20	Medial condyle	Microfracture	0 to 130	0 to 130
5	50	Male	3	Right	20	Medial condyle	Shaving	0 to 120	0 to 130
6	56	Male	4	Right	20	Medial condyle	ACL rec. minor medial meniscus resection Microfracture	0 to 130	0 to 130
7	49	Male	4	Left	17	Medial condyle	Microfracture	0 to 135	0 to 135
8	62	Male	4	Left	17	Medial condyle	Microfracture	0 to 135	0 to 135
9	50	Female	3	Left	17	Medial condyle	Shaving	0 to 135	0 to 135
10	51	Male	3	Right	20	Medial condyle	Microfracture	0 to 140	0 to 140

Ten patients, three females and seven males. ICRS grade 3 or 4 focal knee chondral defect on medial femoral condyle. Median age at surgery 53 years

### Clinical and radiologic assessment

Two patients, 3 and 5, experienced limitations in range of motion. Patient no 3 had a limitation in both extension and flexion, while patient no 5 had a slight flexion deficit (Table 1). Patient no 3 had slight hydrops at examination. No effusion was found in any of the other patients.

One patient, no 2, had a reoperation, a second-look arthroscopy, 10 months after primary surgery due to persistent anterior knee pain. The arthroscopy revealed slight patellofemoral degenerative changes, but the implant was well-fixed and no further surgery has been performed in this or any of the other patients during the follow-up period.

All the patients had normal load-bearing knee X-rays on the contralateral side. Nine patients had normal load-bearing knee X-rays on the operated side. In one patient, pat no 3 with a follow-up of 84 months, the knee X-ray was classified as Ahlbäck 1. This patient has had intermittent problems with effusion and also underwent an X-ray 48 months after index surgery that was also classified as Ahlbäck 1. No progression of osteoarthritis was thus seen between the 48- and 84-month follow-ups.

### Patient-related outcome measurements

**Fig. 1:**
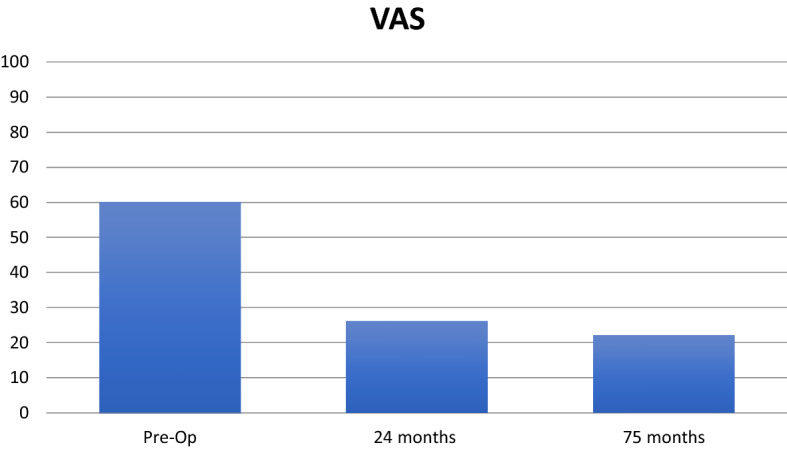
VAS at pre-op, 24 months and the last follow-up at a mean of 75 months. A significant decrease between pre-op and 24 months (*p* < 0.001) was seen, but there was no difference between 24 and 75 months

The median Tegner score was pre-op 3 (1–5), 24 months 4 (3–5) and at 75 months 4 (2–6). The improvement between pre-op and 24 months Tegner reached significance (*p* = 0.034). The EQ5D VAS improvement did not reach significance (Table 2). The VAS for pain decreased significantly between pre-op and the follow-up at 24 months (*p* < 0,001). No further reduction in pain experience was seen between 24 and 75 months and some pain still persisted at the latest follow-up (Fig. 1, Table 2).

**Table 2:** Patient-related outcome measurements from pre-op to the latest follow-up

	EQ5D VAS	VAS	KOOS Pain	KOOS Symptoms	KOOS ADL	KOOS Sports	KOOS QoL
Pre-op	64 (29)	60 (16)	60 (15)	73 (18)	66 (18)	23 (18)	28 (17)
24 months	83 (11)	26 (25)	78 (17)	83 (12)	88 (13)	48 (27)	49 (18)
75 months	81 (13)	22 (18)	85 (11)	82 (14)	91 (9)	48 (19)	55 (21)
Pre-op vs 24 m	*p* = 0.088	*p* < 0.001	*p* = 0.071	*p* = 0.201	p = 0.0048	p = 0.034	p = 0.037
Pre-op vs 75 m	*p* = 0.12	*p* < 0.001	*p*= 0.01	*p* = 0.234	*p* = 0.003	*p* = 0.024	*p* = 0.003
24 m vs 75 m	*p* = 0.592	*p* = 0.697	*p* = 0.392	*p* = 0.747	*p* = 0.478	*p* = 0.850	*p* = 0.542

Data expressed as the mean (standard deviation)

The KOOS subscores for ADL, Sports and Quality of Life improved significantly from pre-op to 24 months [KOOS ADL pre-op mean (SD) 66 (18) vs 24 months 88 (13) *p* = 0.0048; Sports and Quality of life pre-op 23 (18) vs 24 months 48 (27) *p* = 0.034] and the improvement was maintained at the latest follow-up, without a significant increase or decrease between 24 months and the latest follow-up. The KOOS Pain score improvement did not reach significance at 24 months, but a further improvement was seen and was significant at the latest follow-up compared with pre-op [KOOS Pain, pre-op mean (SD) 60 (15) vs 75 months 85 (11) *p* = 0.01], No significant improvements were seen for the KOOS Symptoms score (Table 2).

## Discussion

The most important finding in this study is that good improvements in subjective outcome measurements are maintained from 24 months to a follow-up after a minimum of 5 years and a mean of 75 months for this customized focal knee prosthesis. No conversion to knee arthroplasty has been necessary and in only one patient was radiographic osteoarthritis, Ahlbäck 1, noted at follow-up.

In previous studies of focal knee-resurfacing systems, a higher grade of osteoarthritis development and a higher risk of a subsequent need for total knee arthroplasty, 23%, were reported within seven years [10]. In this study, the follow-up time was slightly shorter, but progression to osteoarthritis was only noted in one patient in ten. In that case, weight-bearing X-rays were normal at the 12-month follow-up. The patient had an extra weight-bearing X-ray done at 48 months due to persistent pain and intermittent effusion that showed Ahlbäck 1 osteoarthritis, but no progression of radiologic degeneration was seen at 84 months. It is known that precise implant positioning is crucial in order not to induce stress on the opposing tibial cartilage and subsequent cartilage degeneration [17]. This customized prosthesis system with patient-specific implants and surgical guide equipment improves the potential for adequate implant positioning, which may explain why this study reports a good result and few patients with osteoarthritis at this mid-term follow-up. However, no perioperative complications were noted in the patient that progressed to osteoarthritis, no second-look arthroscopy was performed and it is not known whether this implant is correctly positioned or what the reason for the progression to osteoarthritis could be. The patient underwent previous surgery with a medial meniscus resection, described as minor, ACL reconstruction and microfracture of the cartilage injury. Careful patient selection is vital. We know that the meniscus is a crucial load-bearing structure, optimizing contact area and minimizing contact stress. The extent and part of the meniscus that is resected is of great importance. A minor flap tear that is resected is probably of limited importance, while root tears cause a loss of hoop tension and an increase in contact stresses similar to total meniscectomy [18]. The meniscus is also an important stabilizer and, in combination with cruciate ligament instability, the risk of developing knee osteoarthritis increases substantially [19, 20]. It is likely crucial to be careful about patient selection and to avoid other risk factors for osteoarthritis, apart from the cartilage injury, when advising the patient to undergo surgery with a focal knee prosthesis. The good results in this study could also be due to caution in patient selection.

The subjective outcome scores improved substantially from pre-operatively to the 2 year follow-up and this improvement was maintained at the latest follow-up at a mean of 75 months post-surgery. The longevity of the prosthesis can therefore be assumed to be good, but it should be noted that some residual pain was still present at the final follow-up. Despite good range of motion, no swelling and no radiologic signs of degenerative changes, some subjective impairment was experienced and this is important to bear in mind when counseling patients. On the other hand, these patients were selected for surgery due to pronounced pain and functional impairment and previous studies of focal cartilage injuries have shown impaired quality of life comparable to that of the severe osteoarthritis in patients on the waiting list for a total knee arthroplasty [3]. Previous biological treatment and rehabilitation were tried and there are few other options for this group of patients with highly symptomatic cartilage injuries. The ways of managing patients who present with failed attempts at the biological treatment of cartilage injuries are not well described in the literature [21]. The symptoms often worsen with time and the degeneration progresses [5].

The main limitations in this study are the small number of well-selected patients and it is not possible from this or other studies of focal knee resurfacing to foresee how the risk for long-term development of osteoarthritis will evolve. But however, even if it is only regarded as a bridge between biological treatment and conventional knee arthroplasty, we can conclude that surgery has the potential to give patients significant pain relief, functional improvement and an increased perception of quality of life for several years after surgery. In the event of failure, the method does not compromise future arthroplasty surgery, as seen in other studies of focal knee resurfacing [10].

## Conclusion

A good subjective outcome, a low risk of progression to degenerative changes and the need for subsequent surgery were seen at the mid-term follow-up with this customized focal knee-resurfacing implant for medial femoral cartilage injuries.
